# Changes in Implant Surface Characteristics and Wettability Induced by Smoking In Vitro: A Preliminary Investigation

**DOI:** 10.3390/ma18122844

**Published:** 2025-06-17

**Authors:** Danielle Ohana, Nina K. Anderson, Rafael Delgado-Ruiz, Georgios E. Romanos

**Affiliations:** 1 Department of Periodontics and Endodontics, School of Dental Medicine, Stony Brook University, Stony Brook, NY 11794, USA; danielle.ohana@stonybrookmedicine.edu; 2 Department of Oral Biology & Pathology, School of Dental Medicine, Stony Brook University, Stony Brook, NY 11794, USA; nina.anderson@stonybrookmedicine.edu; 3 Department of Prosthodontics and Digital Technology, School of Dental Medicine, Stony Brook University, Stony Brook, NY 11794, USA; rafael.delgado-ruiz@stonybrookmedicine.edu

**Keywords:** hydrophilicity, tobacco smoking, wettability

## Abstract

The biologic response following the insertion of dental implants is a widely studied process. Recent research has highlighted the importance of implant surface topography and chemistry as highly influential factors in consolidating the dental implant with the surrounding biological environment. The hydrophilicity, or wettability, of dental implants plays a pivotal role in these interactions and successful osseointegration. A more well-established factor that can also influence the development of the tissue–implant interface is exposure to tobacco smoke. While the negative impact of smoking on the biological response of the tissue is clear, there has been no research evaluating the impact that tobacco smoke can have directly on the surface chemistry of dental implants. The present study aimed to explore the effect of smoking on implant surface chemistry and wettability in vitro. Five different implant disks (Ti-Mach, Ti-SLA, Ti-Alloy, Zirc-1 and Zirc-2) were subjected to contamination with tobacco smoke using a portable smoke infuser with dome enclosure. Occasional smoking (5×/day 10 min each for 3 days) and heavy smoking (20×/day for 10 min each for 10 days) were simulated. The wettability of the implant disks was evaluated via the contact angle technique using artificial blood and albumin, as well as saline as a control. It was determined that the contamination of implant surfaces due to smoking produces changes in the surface chemistry and wettability. Changes in the surface hydrophilicity differed based on the implant material. Within the constraints of this investigation, tobacco smoke improved the hydrophilicity of titanium surfaces but worsened that of ceramic surfaces when utilizing the testing solutions. Different implant surfaces exhibit different wetting behavior following contamination with nicotine smoke. This might have an impact on the treatment of peri-implantitis in smokers due to changes in implant surface hydrophilicity, which can affect the re-osseointegration process.

## 1. Introduction

Surface wettability is regulated by properties such as the surface chemistry and surface topography [1]. Wettability is an important attribute of a dental implant as it determines the series of biological events occurring at the implant and host interface. Essentially, the wetting behavior of the implant will play a pivotal role in the biological response of the surrounding bone and soft tissue [1]. Variations in implant surface energy have been proven to influence early wound healing and affect overall clinical performance [2]. In an in vivo model, the surface energy of an implant was found to affect gene expression in the surrounding tissues [2].

Wettability is typically quantified via the measurement of contact angles, which is the angle between the horizontal solid’s surface and the tangent line to the liquid drop’s surface at the three-phase boundary [3]. We consider wettability favorable, or hydrophilic, when a contact angle produced on a surface is less than 90°, and hydrophobic when above. While the ideal contact angle measurement for optimal biological and clinical outcomes is still unknown, a hydrophilic surface will optimize osseointegration. Better wettability creates ideal conditions for protein adsorption to the dental implant surface, and successful corresponding interactions between those proteins to cell surface receptors. Less wettable surfaces do not only impede these processes, but they are also at risk for hydrocarbon contamination, creating further barriers between the dental implant and the consolidating biological environment [3]. Wettability will directly affect the spreading ability of cells on the surface of dental implants, a pivotal factor in cell adhesion behavior and the occurrence of protein attachment sites [4]. It has been previously established that superhydrophobic surfaces will have a low percentage of surviving cells [4]. Therefore, to optimize the rate and degree of osseointegration and create a favorable biological outcome, the wettability of a dental implant must be a clinical consideration.

While wettability is an important property of dental implants, there is a lack of published data considering the effects of modifications to surfaces on wetting behavior. This gap in the literature is further emphasized when trying to understand the wetting behavior of ceramic implants, as opposed to titanium. The aim of this study was to expand on this need, and address how the wetting behavior and the contact angles of implant surfaces, both ceramic and titanium, changes in response to exposure to tobacco smoke. Although the negative effects tobacco smoke can have on systemic wound healing is clear [5], there are no previous studies on how smoke residues or condensates can affect the dental implant itself. The physiochemical alteration of the dental implant is a factor that must be considered alongside systemic effects to fully understand the re-osseointegration around implants exposed to tobacco smoke. The chemical investigation of tobacco has previously established it as a porous substance containing some hydrophilic colloid materials and water-soluble crystals [6]. Some physiochemical characteristics of tobacco smoke when in contact with dental implants were therefore investigated.

## 2. Materials and Methods

### 2.1. Materials

Dental implant surfaces made of both titanium and zirconia in the form of disks were utilized in this study. Three titanium disks with differing surface treatments or compositions were included: a titanium alloy grade 5 disk (6% aluminum and 4% vanadium), a machined titanium disk, and a sandblasted large-grit acid etched (SLA, Aalberts Surface Technologies GmbH, Lübeck, Germany) titanium disk. Two zirconia surfaces from differing manufacturers were utilized. Strict handling and storage procedures were used to reduce the possibility of sample contamination. Following the modifications described below, every disk was promptly put into sealed, individually labeled containers to avoid cross-contact and environmental exposure. Once ready for use, gloves and sterilized instruments were used for all handling. Sample labeling was standardized and coded to minimize operator bias during subsequent analysis.

### 2.2. Contact Angle Determination

The wettability of each implant disk was evaluated using contact angle measurements. Contact angles were obtained with the sessile drop technique using a contact angle goniometer (error ± 1) and accompanying software* (Ossila® v3.0.6.0 software). Upon calibration, 10 µL of the testing solution was placed on the implant disk. The goniometer was used to record the contact angle of the droplet upon first contact with the implant surface. Implant disk surfaces were tested before and after exposure to tobacco smoke. Results are representative of the mean of 15 trials for each group (*n* = 15).

### 2.3. Occasional Smoking, Primary Effects of Nicotine

Dental implant surfaces of machined titanium (Ti-Mach), titanium SLA (Ti-SLA), and zirconia (Zirc-1^†^) in the form of disks were modified to represent those of occasional smokers, as characterized by Pulvers et al. (2013) [7]. Exposure to tobacco^‡^ smoke for 3 days, 5×/day, for 10 min was performed using a portable smoke infuser with dome enclosure. The setting of smoke infusion and temperature during the exposure of disks was standardized and uniform across sample preparation. The tobacco contained a mixture of Danish Black Cavendish, Brown Cavendish and delicate Virginia tobacco. The wettability was evaluated via the contact angle technique using artificial blood and saline as a control. Each contact angle measurement utilized a distinct unused disk. In total, including the control disks before exposure to tobacco smoke, 180 contact angle measurements were completed, with *n* = 15 per group. Comparisons were completed with the paired *T*-test. Statistical significance was set as *p* < 0.05.

### 2.4. Heavy Smoking

**Chart 1:**
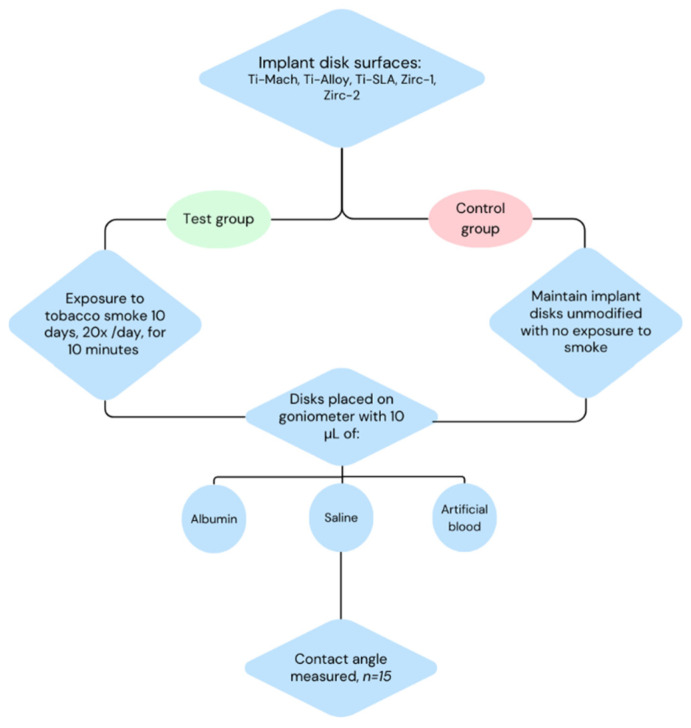
Experimental flow.

Following the pilot study on occasional smoking, additional disks were modified to represent those of heavy smokers [7]. Dental implant surfaces of machined titanium (Ti-Mach), titanium SLA (Ti-SLA), titanium alloy (Ti-Alloy), zirconia (Zirc-1^†^), and zirconia (Zirc-2^§§^) were modified to represent those of heavy smokers, with exposure to tobacco smoke for 10 days, 20×/day, for 10 min using the same technique. The wettability was evaluated via the contact angle technique using artificial blood, albumin^†^ (10 mg/mL in PBS), and saline as a control. These testing solutions were included as they are clinically relevant in simulating the interactions of biological processes near implant sites. In total, including the control disks before exposure to tobacco smoke, 450 contact angle measurements were completed, with *n* = 15 per group. A flow chart of this experimental setup can be visualized in Chart 1. Comparisons were completed with the paired *T*-test. Statistical significance was set as *p* < 0.05.

### 2.5. Surface Roughness Analysis

Four representative specimens (2 titanium: Ti-Mach and Ti-SLA; 2 zirconia: Zirc-1 and Zirc-2) were evaluated before and after both light and heavy smoking simulations using 3D laser confocal scanning microscopy. The surface roughness parameters Sa (arithmetical mean height), Sz (maximum height), and Sdr (developed interfacial area ratio) were recorded for each condition.

### 2.6. CRIS Guidelines

This study adhered to CRIS guidelines, which is the checklist for reporting in vitro studies. The CRIS guidelines evaluate the reporting of the sample size calculation, difference between groups, sample preparation and handling, allocation of samples, and statistical analysis [8].

## 3. Results

### 3.1. Occasional Smoking

**Figure 1:**
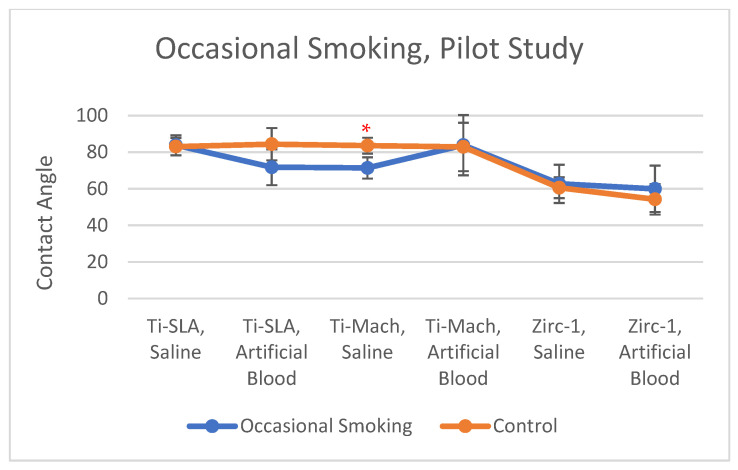
Comparison of contact angles on test implant surfaces exposed to occasional smoking and control surfaces using saline and artificial blood (* signifies *p* < 0.05).

The results of the surface contact angle measurements (wettability test) are displayed in Figure 1. There were no significant differences in wettability between the implant surfaces Ti-SLA and Zirc-1 modified with smoke contamination and the controls. The wettability of the machined titanium surfaces coated with saline significantly increased after occasional smoking but remained similar when evaluated with artificial blood. The wettability of zirconia Zirc-1 implant surfaces is more favorable when compared to Ti-SLA and Ti-Mach implant surfaces. Overall, there was no case in which contamination with nicotine (in occasional smoking) significantly decreased the wettability of the implant surface.

### 3.2. Heavy Smoking

Following the pilot study, the experimental groups were expanded to test additional implant surfaces and solutions. Trials included modified and control surfaces of machined titanium (Ti-Mach), titanium SLA (Ti-SLA), titanium alloy grade 5 (Ti-Alloy), zirconia (Zirc-1), and zirconia (Zirc-2). Wettability was evaluated with artificial blood, albumin, and saline as a control.

**Figure 2:**
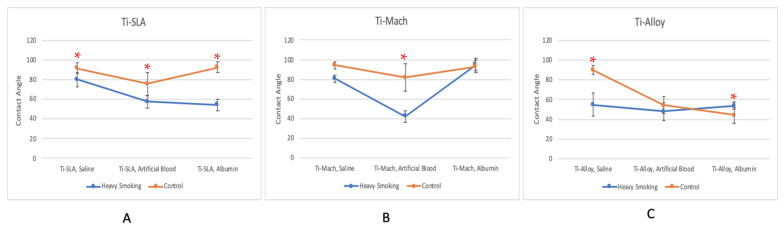
Comparison of heavy-smoking-modified test disk (blue) and control disk (orange) on titanium implant surfaces. (**A**) Sandblasted large-grit acid etched (SLA) titanium disk. (**B**) A machined titanium disk. (**C**) Titanium alloy grade 5 disk (6% aluminum and 4% vanadium). Each respective implant surface was evaluated for contact angles using saline, artificial blood, and albumin (* signifies *p* < 0.05).

Figure 2A displays the wettability of the Ti-SLA surface, in which the modified implant surface produced significantly smaller contact angles when compared to the control, non-modified disk. Therefore, the Ti-SLA surface exhibited significantly better wettability following modification with the heavy smoking protocol using all three testing solutions. Figure 2B represents the results using the Ti-Mach surface, where the significantly better wettability of modified surfaces was observed using artificial blood. There was no significant difference when comparing the modified and control Ti-Mach surfaces using albumin and saline. Ti-Alloy also exhibited better wettability on the modified surface when using saline, as seen in Figure 2C. However, the non-modified control Ti-Alloy disk exhibited significantly better wettability when using albumin. There was no significant difference when comparing modified and control Ti-Alloy surfaces using artificial blood.

**Figure 3:**
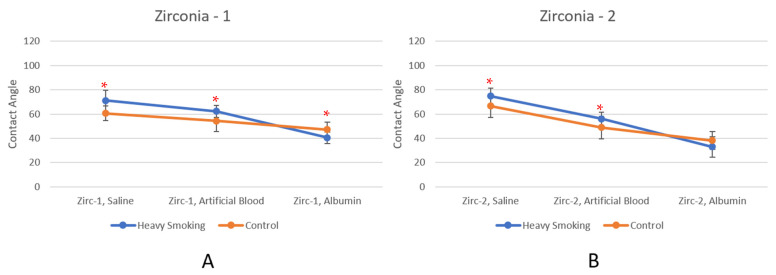
Comparison of heavy-smoking-modified test disk (blue) and control disk (orange) on zirconia implant surfaces. (**A**) Zirc-1 surface, (**B**) Zirc-2 surface. Each respective implant surface was evaluated for contact angles using saline, artificial blood, and albumin (* signifies *p* < 0.05).

When further evaluating the ceramic surfaces, we observed a different wetting behavior compared to the titanium surfaces. Zirconia (Zirc-1) surfaces contaminated with heavy smoke showed significantly lower hydrophilicity when compared to the control using saline and artificial blood, as seen in Figure 3A. The wetting behavior of the contaminated surface when evaluated with albumin differed and was significantly better when compared to the control. Figure 3B represents the wettability of another zirconia surface (Zirc-2). The wettability of the surfaces modified with heavy smoke was significantly worse when compared to the control when tested with albumin and saline. There were no significant differences in wettability between the modified Zirc-2 surface and control when using albumin.

**Figure 4:**
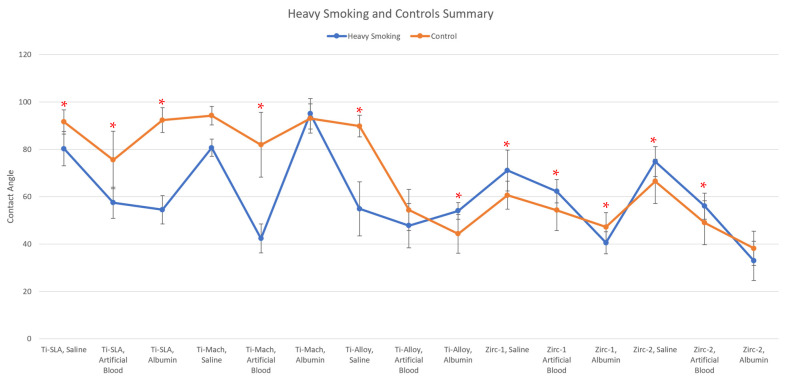
Comparison of contact angles on all test implant surfaces exposed to heavy smoking (blue) and control surface (orange) using saline, artificial blood, and albumin (* signifies *p* < 0.05).

A summary of all heavy smoking trials and comparisons with the controls are displayed in Figure 4. We observed that overall, when using artificial blood and saline as testing solutions, the wetting behavior of titanium implants surfaces improved after modification with heavy smoking, while it worsened for zirconia surfaces. Trials completed using albumin yielded stochastic results. When comparing the control surfaces among different dental implant materials, Ti-Alloy, Zirc-1, and Zirc-2 exhibited more favorable wetting behavior when compared to the Ti-SLA and Ti-Mach surfaces. A summary table with all measured contact angles and percent changes from control values is displayed in Table 1. A negative % change, as indicated by titanium surfaces when using artificial blood and saline as testing solutions, represents a lower contact angle after the modification of the surface, meaning better wettability. The opposite, or a positive % change, is displayed with the corresponding zirconia results, indicating worsened wettability after modification with heavy smoking.

### 3.3. Surface Rougness Analysis

The results of the surface roughness analysis are displayed in Table 2. Zirconia surfaces (Zirc-1, Zirc-2) and machined titanium (Ti-Mach) exhibited the lowest Sa and Sz values, indicating smoother baseline topographies. Light and heavy smoking resulted in slight increases in Sa and Sz across all surfaces, suggesting minor surface roughening. However, the Sdr values—representing the developed surface area ratio—remained relatively stable compared to baseline measurements. The results demonstrate minimal to negligible changes in roughness parameters following exposure to smoking protocols. These findings suggest that while tobacco smoke may introduce surface chemical modifications or adsorptive films, it does not appreciably alter the surface topography of the tested implant materials under the conditions of our study, as the surface texture was not significantly altered by smoke exposure. This supports the hypothesis that observed wettability changes are likely due to surface chemistry (e.g., thin film residues), rather than macro- or micro-topographic alterations.

## 4. Discussion

To understand the importance of tobacco smoke’s influence on implant surfaces, it is pivotal to recognize the effect smoking has on the oral cavity and beyond. Exposure to tobacco smoke is a prevalent issue, with adverse effects on both systemic and oral health. Specifically, in the oral cavity, smoking has been proven to be a risk factor for periodontal disease, increasing plaque accumulation, the resorption of alveolar bone, and hindering host immune response [9]. In consideration of overall health, smoking has been linked to delayed wound healing by decreasing blood flow and therefore tissue oxygenation [9]. The components of tobacco smoke, primarily nicotine and its metabolites, affect the endothelium, blood protein attachment, and coagulation [10].

Many studies have been performed to identify the exact effect of nicotine on these properties of wound healing and serum behavior [11,12,13]. While smoking impairs endothelial function, it is notable that it increases platelet adhesiveness and aggregation, fibrinogen levels, and plasma viscosity [10]. Platelets then participate in the production of an inflammatory response, depositing pro-inflammatory cytokines, growth factors, and other inflammatory mediators [14]. The coagulation–fibrinolysis cascade is impaired at multiple levels, often leading to thromboembolic events of blood vessels, and in more severe cases, the heart and brain [10].

With these dire consequences in mind, it is important to consider the effect of nicotine not only on coagulation, but on the hemostatic process and blood protein morphology [9]. Smoking has been proven to change the fibrin network ultrastructure. This has been previously described by Pretorius et al. as the Sticky Fibrin Phenomenon [15]. Fibrin networks are affected by exposure to tobacco smoke as they arrange in thickened, plaque-like areas. These create large areas of sticky fibrin, which cannot be broken down by fibrinolysis [15]. This may serve as an explanation for the stickier, thicker appearance of blood observed clinically in smoking patients.

A specific analysis of peri-implant tissue showed that it is the local absorption of tobacco smoke components that has a primary influence on post-operative healing, as opposed to systemic effects [9]. Knowing the impact of tobacco smoke, mainly nicotine, on blood protein attachment, this study performs a preliminary investigation to find if these properties can be translated to implant surfaces. While the role of the host response to smoking as a risk factor for implant failure is clear, the effect on the implant surface itself and local integration is not. The consequences of tobacco smoke on implant wettability and surface chemistry were evaluated, in order to determine changes in the hydrophilicity of dental implants. The improved wettability of titanium surfaces after modification with tobacco smoke might relate to these principles.

In addition to changes in wettability, exposure to tobacco smoke can also influence the physical and mechanical properties of dental implant surfaces. Indeed, tobacco smoke contains a variety of organic compounds, heavy metals, and fine particulates that can adhere to implant materials and alter their surface morphology and chemical composition [16].

On titanium implants, these contaminants may interfere with the integrity of the naturally occurring titanium oxide layer (TiO₂), which is crucial for corrosion resistance and biocompatibility [17]. Meanwhile, on zirconia implants, the contamination produced by tobacco smoke could affect the zirconia oxide layer, alter the color, and promote low-temperature degradation [18,19]. Furthermore, any changes in surface chemistry and topography can influence how proteins adsorb to the material, how cells attach and spread, and how tissues integrate with the implants [20].

To the author’s knowledge, this is the first report investigating the wettability of dental implant surfaces after exposure to tobacco smoke. Previous studies have investigated tobacco-mediated adhesion to soft tissues and biofilms [21,22,23]. Bacteria exposed to cigarette smoke condensate (CSC) were shown to adhere more to epithelial cells compared to non-exposed specimens, suggesting that components in tobacco smoke influence epithelial colonization by microbes [21,22]. Baboni et al. (2010) found that the exposure of orthodontic materials to CSC can enhance cariogenic and *Candida* biofilm formation [23]. Specifically, they observed a positive influence on the adhesion of *S. mutans* and *C. albicans* to brackets, bands, and acrylic resin stripes exposed to CSC [23]. Previous literature suggests that many rigid intraoral surfaces have an acquired pellicle that can interfere with microbial adhesion [24]. It is plausible that CSC annihilates these repulsive properties and creates a more hydrophilic surface that contributes to increased adhesion. CSC promoting the cellular adhesion of *C. albicans* with a linear dose response was seen in several other studies [25,26]. While these investigations can be associated with changes to the intraoral surfaces that promote cellular adhesion, it is important to consider the infection-promoting properties this exposure can have in a clinical application.

A deeper analysis of the chemical composition of tobacco smoke or cigarette smoke condensate can clarify the results obtained. Previous studies have established that the majority of the components of tobacco smoke residue arise from the tobacco itself, not artifacts that may form during the processing of the smoke condensate [27]. Tobacco, and specifically nicotine, have been shown to have the ability to exist in both non-aqueous and water-soluble phases [27]. The phase in which the chemical constituents remain can be influenced by many factors, including the polarity and porosity of the surface onto which the tobacco smoke settles. The authors hypothesize that the difference in wettability between the two types of dental implant surfaces, titanium and zirconia, with a tobacco smoke film can be due to inert differences in surface polarity. Finally, while assessing the effects of tobacco smoking on the mechanical properties of titanium and zirconia disks was beyond the scope of the present work, future studies should consider if exposure to tobacco smoke compromises mechanical properties including fracture toughness, fatigue resistance, or surface hardness.

Ultimately, the investigation of wetting behavior (hydrophilicity) should help draw conclusions regarding the consolidation of dental implants with the surrounding biological environment after exposure to tobacco smoke. Understanding the differences in the wetting behavior of titanium and zirconia implants using different solutions has important clinical implications, especially when peri-implant defects due to inflammation have to be debrided and grafted with grafting materials. Further investigation can help create recommendations for re-osseointegration, particularly in cases of peri-implant defects with exposed implant threads in patients who are smokers and susceptible to developing the tobacco film described prior to treatment.

It is important that we state that this study only focuses on wettability testing using potential treatment solutions. The solutions used were ones that the authors felt are clinically relevant to simulate the interactions of biological processes during re-osseointegration. This is not reflective of the usual protocol established for wettability tests. The use of albumin in this study yielded high variability and therefore did not contribute to the reliability of results. Additional limitations include the small sample size included, as the statistical power was diminished. A greater number of repetitions with expansion using additional testing solutions and a blinded assessment model would have strengthened results. Further studies might focus on an established wettability evaluation protocol, or incorporate grafting materials in conjunction with these solutions, in order to more accurately assess the impact of wettability on the re-osseointegration of these implant surfaces. Moreover, additional factors evaluating surface characteristics beyond wettability were not included. Further investigation could focus on additional analyses to test the functional impact of the surface changes, such as protein adsorption studies, cellular attachment, or biofilm formation. These studies will better represent how changes to the surface affect the re-osseointegration process and host response within the oral cavity. Future research can also expand the sample size to create an improved statistical design and increase the robustness of the conclusions. Lastly, the study possesses a moderate risk of bias, with a lack of blinded assessments.

## 5. Conclusions

The contamination of implant surfaces due to smoking produces changes in contact angles and wettability of the implant surfaces. Different implant surfaces exhibit different behaviors of surface wettability. Within the limitations of this study, tobacco smoke improves the hydrophilicity of titanium surfaces but creates hydrophobic behavior on ceramic surfaces when testing with saline and artificial blood.

**Vendor List**

- Ossila, Sheffield, UK.
- Swiss Dental Solutions, Konstanz, Germany.
- W.O. Larsen Lotus pipe aromatic tobacco 100 g flavored by cocco, vanilla, Copenhagen, Denmark.
- Lampire Biological, Pipersville, PA, USA.
- Patent, Altendorf, Switzerland.

## Figures and Tables

**Table 1:** Summary table with all measured contact angles and % changes from control values.

Disk, Test Solution	Heavy Smoking (˚)	Control (˚)	% Change	*p* < 0.05
Ti-SLA, Saline	80.25	91.59	−12.38%	Yes
Ti-SLA, Artificial Blood	57.45	75.50	−23.91%	Yes
Ti-SLA, Albumin	54.46	92.35	−41.03%	Yes
Ti-Mach, Saline	80.66	94.27	−14.44%	No
Ti-Mach, Artificial Blood	42.34	81.93	−48.32%	Yes
Ti-Mach, Albumin	95.02	93.04	2.12%	No
Ti-Alloy, Saline	54.85	89.83	−38.94%	Yes
Ti-Alloy, Artificial Blood	47.77	54.35	−12.12%	No
Ti-Alloy, Albumin	54.02	44.30	21.96%	Yes
Zirc-1, Saline	71.08	60.57	17.36%	Yes
Zirc-1 Artificial Blood	62.25	54.26	14.74%	Yes
Zirc-1, Albumin	40.51	47.19	−14.15%	Yes
Zirc-2, Saline	74.83	66.47	12.57%	Yes
Zirc-2, Artificial Blood	56.01	49.00	14.32%	Yes
Zirc-2, Albumin	32.92	38.16	−13.74%	No

**Table 2:** Surface roughness parameters (Sa, Sz, and Sdr) for titanium and zirconia implant surfaces before and after exposure to light and heavy smoking.

Material	Smoking Exposure	Sa (µm)	Sz (µm)	Sdr (%)
**Ti-Mach**	Before	0.72	4.52	5.98
**Ti-Mach**	Light Smoking	0.74	4.55	6.00
**Ti-Mach**	Heavy Smoking	0.75	4.58	6.10
**Zirc-1**	Before	0.52	3.37	2.48
**Zirc-1**	Light Smoking	0.53	3.42	2.56
**Zirc-1**	Heavy Smoking	0.54	3.45	2.64
**Ti-SLA**	Before	1.23	7.59	8.74
**Ti-SLA**	Light Smoking	1.22	7.55	8.71
**Ti-SLA**	Heavy Smoking	1.25	7.64	8.83
**Zirc-2**	Before	0.60	3.58	3.04
**Zirc-2**	Light Smoking	0.61	3.59	3.12
**Zirc-2**	Heavy Smoking	0.63	3.61	3.20

## Data Availability

The original contributions presented in the study are included in the article, further inquiries can be directed to the corresponding author.
